# Machine learning clinical prediction models for acute kidney injury: the impact of baseline creatinine on prediction efficacy

**DOI:** 10.1186/s12911-023-02306-0

**Published:** 2023-10-09

**Authors:** Amir Kamel Rahimi, Moji Ghadimi, Anton H. van der Vegt, Oliver J. Canfell, Jason D. Pole, Clair Sullivan, Sally Shrapnel

**Affiliations:** 1 Queensland Digital Health Centre, Faculty of Medicine, The University of Queensland, Herston, Brisbane, 4006 Australia; 2 Digital Health Cooperative Research Centre, Australian Government, Sydney, NSW Australia; 3 The School of Mathematics and Physics, The University of Queensland, St Lucia, Brisbane, 4072 Australia; 4 UQ Business School, The University of Queensland, St Lucia, Brisbane, 4072 Australia; 5 Dalla Lana School of Public Health, The University of Toronto, Toronto, Canada; 6 ICES, Toronto, Canada; 7 Metro North Hospital and Health Service, Department of Health, Queensland Government, Herston, Brisbane, 4006 Australia

**Keywords:** Artificial intelligence, Machine learning, Acute kidney injury, Decision Support System, Clinical, Alert fatigue, Health personnel

## Abstract

**Background:**

There are many Machine Learning (ML) models which predict acute kidney injury (AKI) for hospitalised patients. While a primary goal of these models is to support clinical decision-making, the adoption of inconsistent methods of estimating baseline serum creatinine (sCr) may result in a poor understanding of these models’ effectiveness in clinical practice. Until now, the performance of such models with different baselines has not been compared on a single dataset. Additionally, AKI prediction models are known to have a high rate of false positive (FP) events regardless of baseline methods. This warrants further exploration of FP events to provide insight into potential underlying reasons.

**Objective:**

The first aim of this study was to assess the variance in performance of ML models using three methods of baseline sCr on a retrospective dataset. The second aim was to conduct an error analysis to gain insight into the underlying factors contributing to FP events.

**Materials and methods:**

The Intensive Care Unit (ICU) patients of the Medical Information Mart for Intensive Care (MIMIC)-IV dataset was used with the KDIGO (Kidney Disease Improving Global Outcome) definition to identify AKI episodes. Three different methods of estimating baseline sCr were defined as (1) the minimum sCr, (2) the Modification of Diet in Renal Disease (MDRD) equation and the minimum sCr and (3) the MDRD equation and the mean of preadmission sCr. For the first aim of this study, a suite of ML models was developed for each baseline and the performance of the models was assessed. An analysis of variance was performed to assess the significant difference between eXtreme Gradient Boosting (XGB) models across all baselines. To address the second aim, Explainable AI (XAI) methods were used to analyse the XGB errors with Baseline 3.

**Results:**

Regarding the first aim, we observed variances in discriminative metrics and calibration errors of ML models when different baseline methods were adopted. Using Baseline 1 resulted in a 14% reduction in the f1 score for both Baseline 2 and Baseline 3. There was no significant difference observed in the results between Baseline 2 and Baseline 3. For the second aim, the FP cohort was analysed using the XAI methods which led to relabelling data with the mean of sCr in 180 to 0 days pre-ICU as the preferred sCr baseline method. The XGB model using this relabelled data achieved an AUC of 0.85, recall of 0.63, precision of 0.54 and f1 score of 0.58. The cohort size was 31,586 admissions, of which 5,473 (17.32%) had AKI.

**Conclusion:**

In the absence of a widely accepted method of baseline sCr, AKI prediction studies need to consider the impact of different baseline methods on the effectiveness of ML models and their potential implications in real-world implementations. The utilisation of XAI methods can be effective in providing insight into the occurrence of prediction errors. This can potentially augment the success rate of ML implementation in routine care.

**Supplementary Information:**

The online version contains supplementary material available at 10.1186/s12911-023-02306-0.

## Introduction

Acute kidney injury (AKI) is characterised by a suddenreduction in kidney function, recognised by an increase in serum creatinine (sCr) or a decrease in urine output [1]. It is reported that up to 45% of Intensive Care Unit (ICU) patients and 20% of hospitalised patients experience AKI [2, 3]. Following an episode of AKI, patients have a higher risk of in-hospital mortality and long-term progression of chronic kidney disease (CKD) and kidney failure (KF) [4, 5]. Hospital-acquired AKI poses a substantial burden in terms of adverse health outcomes including extended hospital stays, increased health costs and increased mortality [6]. Early detection of AKI plays a key role in guiding effective therapeutic intervention in hospital settings [7, 8].

AKI has been variously defined over past decades, ranging from the RIFLE classification (Risk, Injury, Failure, Loss of kidney function, and End-stage renal disease), the AKIN criteria (Acute Kidney Injury Network) and the KDIGO guidelines (Kidney Disease Improving Global Outcome) [9–11]. Currently, the latter is accepted as the gold standard for AKI definition within the nephrology community [12]. According to the KDIGO guidelines, AKI is diagnosed either from a rise in the sCr by 26.5 umol/l within 48 h or by an increase of 1.5 times from the baseline sCr within 7 days. It is classified into stages by severity based on the magnitude of changes in sCr or urine output. The most reliable estimation of creatine baseline is presumed to be the mean preadmission sCr value 7 to 365 days before hospitalisation [2, 13], nevertheless, this is often missing in the clinical data of hospitalised patients [14]. One proposed way to address this is by backward calculation of baseline sCr using the Modification of Diet in Renal Disease (MDRD) formula, assuming an estimated glomerular filtration rate (eGFR) value of 75 ml/min/1.73 m^2^ [12, 15, 16].

The estimation of baseline sCr has been important in studies developing and validating Machine Learning (ML) models to predict AKI [17]. The analysis of the papers identified in a recent systematic review revealed that at least 27 AKI studies used 18 variations of baseline sCr to establish the ground truth in order to label positive AKI occurrences [18]. While the baseline sCr serves as a reference point to estimate AKI, it remains unclear whether the variations in estimating this value can confound the comparability of these models. This variation may also lead to a poor understanding of implemented model effectiveness and the potential risks this may pose in clinical workflows.

Alert fatigue is a significant concern in routine clinical workflows when an overwhelming volume of false alarms is generated by decision support systems (CDSSs) [19]. When clinicians and end-users are exposed to this excessive number of warnings, alert fatigue may occur resulting in desensitisation to alarms and an increased likelihood of missed alarms [20, 21]. The precision metric evaluates the proportion of positive predictions made by the classifier that are incorrect. This metric is particularly crucial because it accounts for the FP events which may contribute to alert fatigue [22]. The recent systematic review found that only 17.4% (8 out of 46) of the AKI studies reported the precision metric with a median value of 0.59 ranging from 0.18 to 0.98 [18]. Of all these 8 studies, four reported a precision lower than 0.50 suggesting a high probability of FP events [23–26]. This warrants further analysis of the FP cohort to provide valuable insights into the underlying factors contributing to alert fatigue, leading to mitigate the risk of these false warnings in future implementation endeavours.

In this paper, we seek to conduct a methodological exploration to investigate the following research questions (RQs):

**Table:** 

RQ1	Does the absence of a standardised baseline sCr method impact the performance of predictions in ML models for early detection of AKI incidence?
RQ2	Can conducting an error analysis of ML models provide insights into underlying factors contributing to the FP events?

In addressing these questions, we aim to:

- Develop a suite of ML models using three methods of baseline sCr to predict AKI cases and assess the performance of models.
- Characterise the FP cohort in our analysis using the Explainable AI (XAI) techniques.

## Materials and methods

### Study design

This study followed the Transparent Reporting of a Multivariable Prediction Model for Individual Prognosis or Diagnosis (TRIPOD) reporting guideline [27] which is presented in Supplementary Material 1. The ICU module of the MIMIC-IV dataset was used for the development of the ML models in this study [28]. We included patients with at least one sCr measurement on days 1, 2 and 3 following ICU admissions. This was to identify all existing AKI incidences on each day and to avoid under-estimation of AKI cases. We estimated AKI cases solely on the sCr criterion of the KDIGO definition defined as an increase in sCr of ≥ 0.3 mg/dL (26.5 μmol/L) within 48 h or a change of ≥ 50% from the sCr baseline within 7 days [12]. According to the recommendations from the KDIGO working group and most recent AKI research with the MIMIC dataset, three methods of baseline sCr were adopted to estimate AKI incidence as per the following:

- *Baseline sCr 1*:  The minimum sCr value within the first 24-h of ICU admission [12]
- Baseline sCr 2: The pre-ICU baseline sCr was defined by using the MDRD backward calculation based on age and gender with an eGFR value of 75 ml/min/1.73 m2 ( sCrGFR-75) [16] to identify and exclude patients with AKI on day 1 of admission. The formula presented below represents the calculation of sCrGFR- 75

.

$${SCr}_{GFR-75}= {\left(\frac{75}{\left[186*\left({age}^{-0.203}\right)*\left(0.742 \left(female\right)\right)\right]}\right)}^{-0.887}$$

We then used the minimum creatine value on day 1 of admission as the baseline SCr. This approach was used by Zimmerman and colleagues [29] to predict AKI cases using the MIMIC dataset.

- *Baseline sCr 3*: The pre-ICU baseline sCr was defined as the mean of all sCr values in the 180 to 7 days before ICU admissions [13, 30]. When baseline sCr was not available, the MDRD backward equation was calculated using age and gender to estimate baseline sCr (sCr_GFR-75_) [16]. Recently published research deployed this approach to predict AKI in ICU patients in the MIMIC dataset [31].

**Fig. 1:**
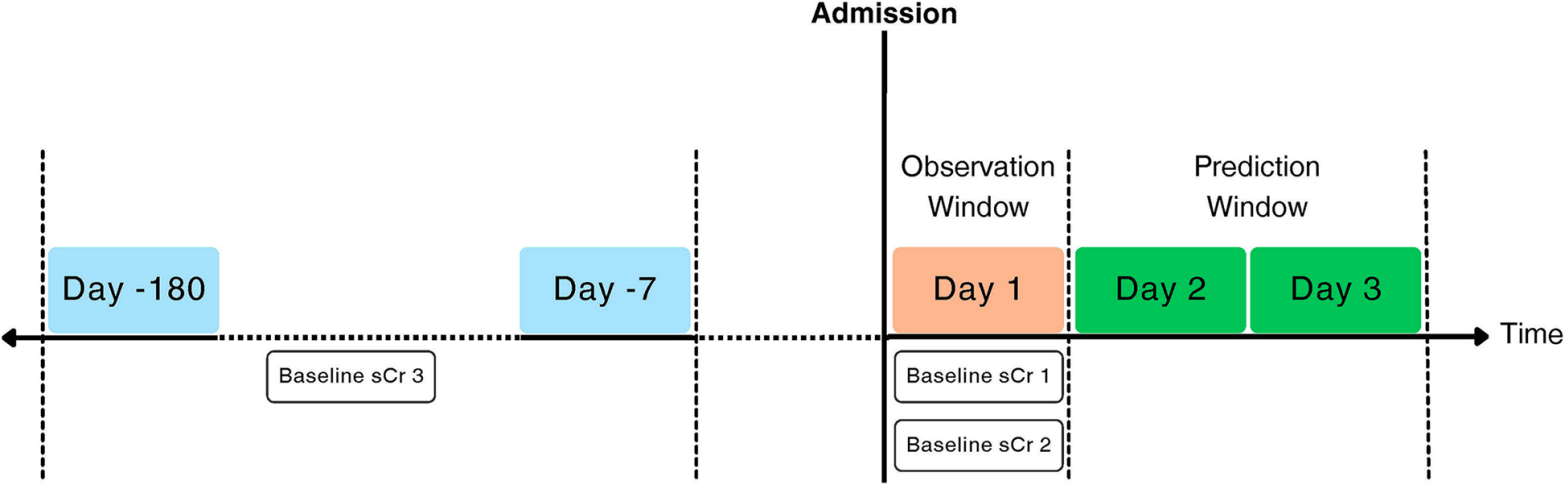
Prediction and observation windows in this study along with the defined methods of baseline serum creatinine. Abbreviation: sCr; Serum Creatinine

Three patient cohorts were constructed using each baseline sCr. Each patient cohort was formed of all the input predictors on the first day of admission and was labelled with the occurrence of AKI on days 2 or 3 as the outcome of the prediction. Figure 1 illustrates the prediction and observation windows for the ML models along with the baseline methods within these windows in this study.

### Input predictors

We extracted candidate categorical and continuous predictors based on previous studies [29, 31–34] and in consultation with subject matter experts in our research team. The candidate predictors in this study were: age at admission to ICU, gender and ethnicity of the patient, vital signs and lab results during the first 24 h of ICU admission, the eGFR by Chronic Kidney Disease Epidemiology Collaboration (CKD-EPI) [35], the use of mechanical ventilation on the first day of ICU admission, past medical history and the average urine output of the first 24 h of ICU admission. The ethnicity variable was categorical and transformed into binary features for each category using one-hot encoding. This was to ensure that there is no ordinal relationship between categories and each category is treated as a separate and distinct feature. We utilised the International Classification of Diseases (ICD) codes to identify patients’ comorbidities. The candidate predictors used in this study, along with the measurement units and relevant ICD codes, can be found in Supplementary Material 2.

### Data aggregation

Patient records were aggregated based on admission ID to include the minimum and maximum of vital signs and laboratory variables during each admission. Each input variable regarding the past medical history was set to 1 when at least one positive record was recorded in the past hospital admissions.

### Exclusion criteria

Only adult patients (age ≥18) were included in the analysis. To avoid any potential treatment-related bias, we only included the first admission to ICU for each patient. Given the difficulties of diagnosing AKI in patients on maintenance renal replacement therapy (dialysis or transplantation), these patients were excluded from the analysis. We recorded the day of AKI detection for each record. The patients with positive AKI on day 1 were excluded because our goal was to predict new AKI cases. The day of AKI detection was also excluded from the final datasets.

### Missing data

Variables with the highest level of missingness included albumin level (68.37%), bilirubin level (50.85%), BMI (48.08%), globulin (98.69%) and thrombin (99.83%). Our analysis relied on a two-stage procedure. First, we excluded the variables with missing values greater than 20% in this study because variables with a large percentage of missingness may not provide enough information for the model to accurately predict the outcome. The counts of missing variables for each input predictor are outlined in Supplementary Material 3. In the second phase, missing data for the remaining variables were imputed using Multiple Imputation by Chains Equations (MICE) method [36].

### Statistical analysis

In this study, we used bootstrapping to randomly sample a subset of data (n = 500) on the test dataset for each baseline method. The f1 scores of the XGB models were calculated for these subsamples. We analysed the variance of these f1 scores to assess the statistical differences. To compare the pairwise differences between the XGB models across three datasets, we performed Tukey's Honesty Significance Difference (HSD) test [37]. The significance level was selected where *p* ≤ *0.05.*

### Development and validation of classification models

All models were developed with the Scikit-learn package (v.1.1.1) in Python language [38]. Four commonly used ML models were developed to predict patients with positive AKI: logistic regression (LR), random forest (RF), eXtreme Gradient Boosting (XGB) and artificial neural network (ANN). To train and evaluate the performance of our ML models, data was randomly split into the train, test and internal validation sets with a ratio of 60/20/20 respectively. A custom Python script was developed to perform hyperparameter tuning on the training set with fivefold cross validation to optimise the f1 score. This is because the f1 score represents a harmonic metric that balances both recall and precision. We used the up-sampling technique on positive AKI cases, but only the training set within each cross-validation fold was used to ensure that the validation and test sets remained intact as unseen data. To ensure consistent scaling across features and prevent the dominance of features with larger values, we performed normalisation on the numerical features, scaling their values within the range of 0.0 to 1.0. For the binary classification where class 0 and class 1 denote no-AKI and AKI respectively, we calculated the predicted probability for each class on the validation and test sets. The predicted probability measures the likelihood of the prediction obtained for that observation in each class, represented as a float value between 0.0 and 1.0.

Model calibration is the process of adjusting the predicted probabilities of a predictive model aiming to align them with the true probabilities within certain intervals—an important effort to avoid any potential harm in CDSSs [39]. While model calibration is an underreported analysis in the prior AKI prediction models [18], we calibrated all models on the validation set and assessed the calibration error metrics on the unseen test sets. Calibration curves were plotted for all models. To evaluate the calibrated models on the unseen test sets, three commonly used calibration error metrics were reported: Estimated Calibration Error (ECE), Brier Score and calibration slope.

Following the calibration, the classification thresholds were selected on the validation sets to optimise the f1 scores. The selected thresholds were used to evaluate ML models on the hold-out test sets, assessing the discriminative metrics of Area Under the Receiver Operating Characteristic Curve (AUC), recall, precision and f1 score.

### Error analysis

We selected the XGB model with the Baseline sCr 3 for this error analysis since this particular baseline method incorporates preadmission sCr, recognised as a reliable baseline approach [2, 13]. Additionally, this model showed a slightly better calibration performance in our analysis. We analysed the errors of this XGB model to gain insight into the decision-making factors of the models and uncover potential causes of error. On the dataset level (global explanations), we used SHAP [40] in Python language. Dalex Python package [41] was utilised to explain the instance-level (local) explanations because of its intuitive representation and comprehensive API documentation. We used the break-down plot which is an intuitive illustration of instance-level predictions and measures the impact of input variables on the outcome of a single prediction. When using the break-down plot to explain a single prediction, it is important to consider that the representation of the plot can differ depending on the ordering of input variables. To ensure the robustness of the results obtained from the break-down plot, we also utilised the SHAP plot within the Dalex framework that computes the average of each single variable across all variables.

## Results

**Fig. 2:**
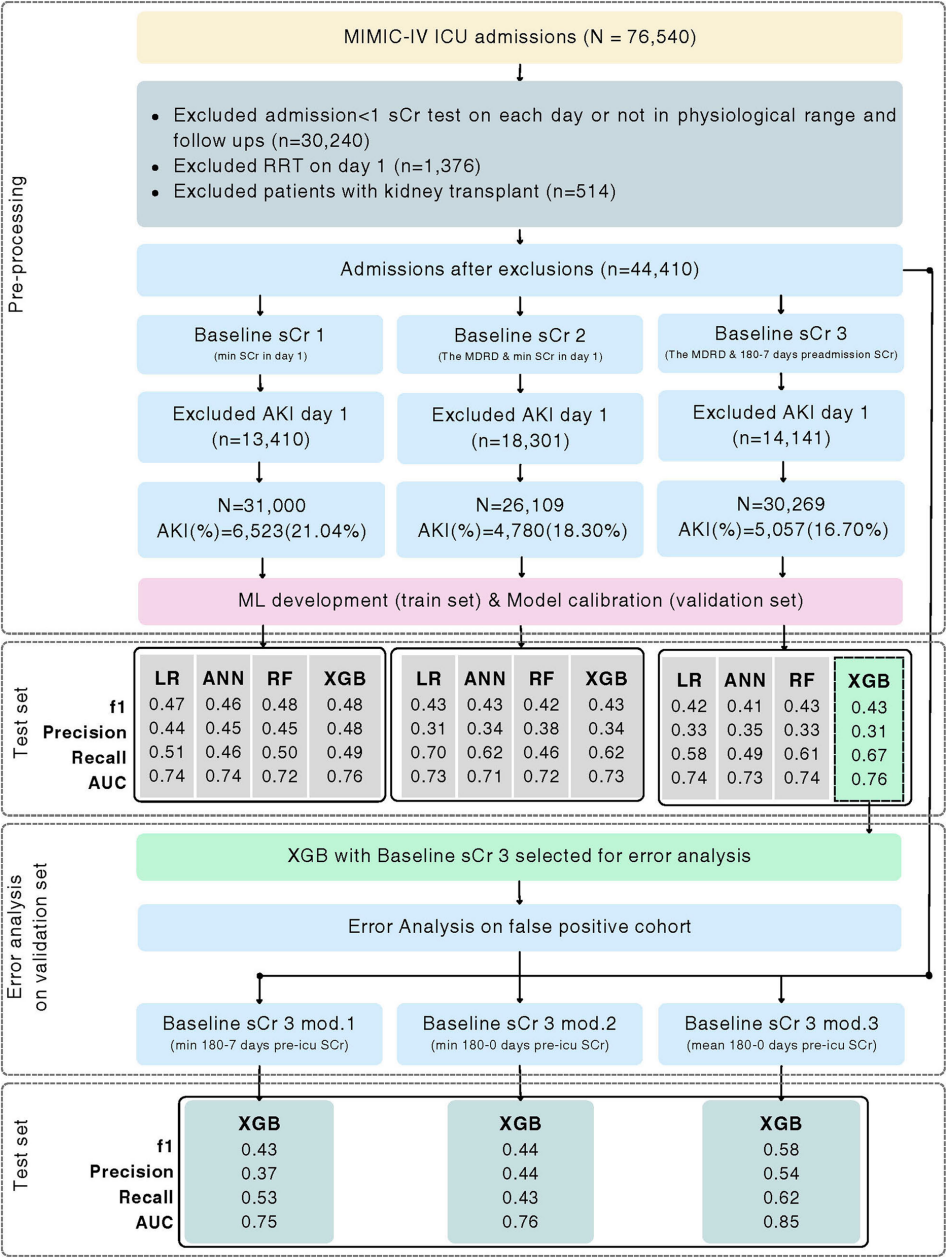
Overall model development flowchart. ANN: Artificial Neural Networks, LR: Logistic Regression, MDRD: Modification of Diet in Renal Disease formula, mod; modified, RF: Random Forest, RRT: Renal Replacement Therapy, sCr; serum Creatinine, XGB: eXtreme Gradient Boosting*.* Abbreviation: ANN, Artificial Neural Networks; Cal. slope, Calibration slope; ECE, Estimated Calibration Error; LR, Logistic Regression; RF, Random Forest; XGB, eXtreme Gradient Boosting

From 76,540 ICU admissions in the MIMIC-IV dataset, 32,130 records did not meet our inclusion criteria and were excluded. In the remaining 44,410 admissions, AKI incidence was estimated using the KDIGO definition with three methods of baseline sCr calculations to construct three patient cohorts. After exclusions, the incidence of AKI in each cohort using Baseline sCr 1, Baseline sCr 2 and Baseline sCr 3 cohorts was 21.04%, 18.30% and 16.70%, respectively. ML models were trained for each cohort. Figure 2 illustrates the overall methods and accuracy metrics of the study.

### RQ1 findings: assessment of the variance between the performance of ML models

**Fig. 3:**
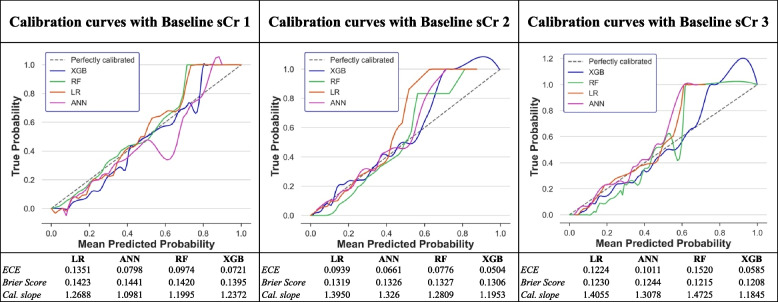
Calibration assessment of the ML models in this study on the test sets

The calibration curves were plotted on the holdout test set across all models displaying differences in calibration errors depending on the baseline methods used (Fig. 3). The calibration curves of the LR and ANN models with Baselines 2 and 3 seem to be roughly aligned to the perfect calibration line when the average predicted probability ranges from 0.0 to 0.4. Most models tend to underestimate AKI incidence when the average predicted probability ranges from 0.6 to 1.0. In general, a smaller value of the ECE and Brier score, along with a calibration slope close to 1.0, indicates better calibration performance. Of all the models in this study, the XGB with the Baseline sCr 3 cohort exhibited predicted probabilities that more closely matched the true probabilities in this cohort, as suggested by an ECE of 0.0585, a Brier Score of 0.1208 and a calibration slope of 1.1845.

The classification threshold was selected to optimise the f1 scores on the validation sets. The performance metrics on the validation sets along with the selected thresholds can be found in Supplementary Material 4. Using the selected threshold, each calibrated model was evaluated on the test set to measure the discriminative metrics (f1, AUC, precision and recall). In our analysis, we observed variations in discriminative metrics among all models with three baseline methods (Fig. 2). The f1 scores for the XGB ranged from 0.43 to 0.50 across all baseline methods while RF ranged from 0.42 to 0.47, ANN from 0.42 to 0.48 and LR from 0.41 to 0.48.

Following the model calibration, we compared the f1 scores of the XGB models across all baselines to assess the statistical differences. XGB models were selected as they showed a slightly better generalisability across all baselines. The HSD test showed that there were statistically significant differences between Baseline 1 and both Baseline 2 (*p* = 0.0311) and Baseline 3 (*p* = 0.0306). The analysis of performance metrics at the selected significance level (0.05) indicates that the adoption of Baseline 1 in this study led to a 14% decrease in the f1 score for both Baseline 2 and Baseline 3. The HSD test also suggested that the differences between Baseline 2 and Baseline 3 were not statistically significant (f1 = 0.43, *p* = 1.0). Overall, these observations indicated that the use of different baseline methods may result in varied estimations of AKI and potentially impact the accuracy of predictions with ML models.

### RQ2 findings: error analysis of the selected model

The XGB with the Baseline sCr 3 method was selected to analyse the prediction errors at both local (prediction instances) and global (model level) explanations. Of all the included 44,410 admissions, the occurrence of AKI was 13,995 (31.51%). The final cohort consisted of 30,269 admissions. A total of 5,057 (16.70%) AKI cases resulted following the exclusion of AKI on day 1. Of all the included admissions in the model development, 17,995 (64.19%) did not have baseline pre-ICU sCr. Among admissions without baseline pre-ICU sCr, AKI was detected in 2,504 (13.91%) using sCr_GFR-75_. For all the remaining 10,035 admissions with baseline pre-ICU sCr, a total of 1,755 (17.48%) were diagnosed with AKI.

**Fig. 4:**
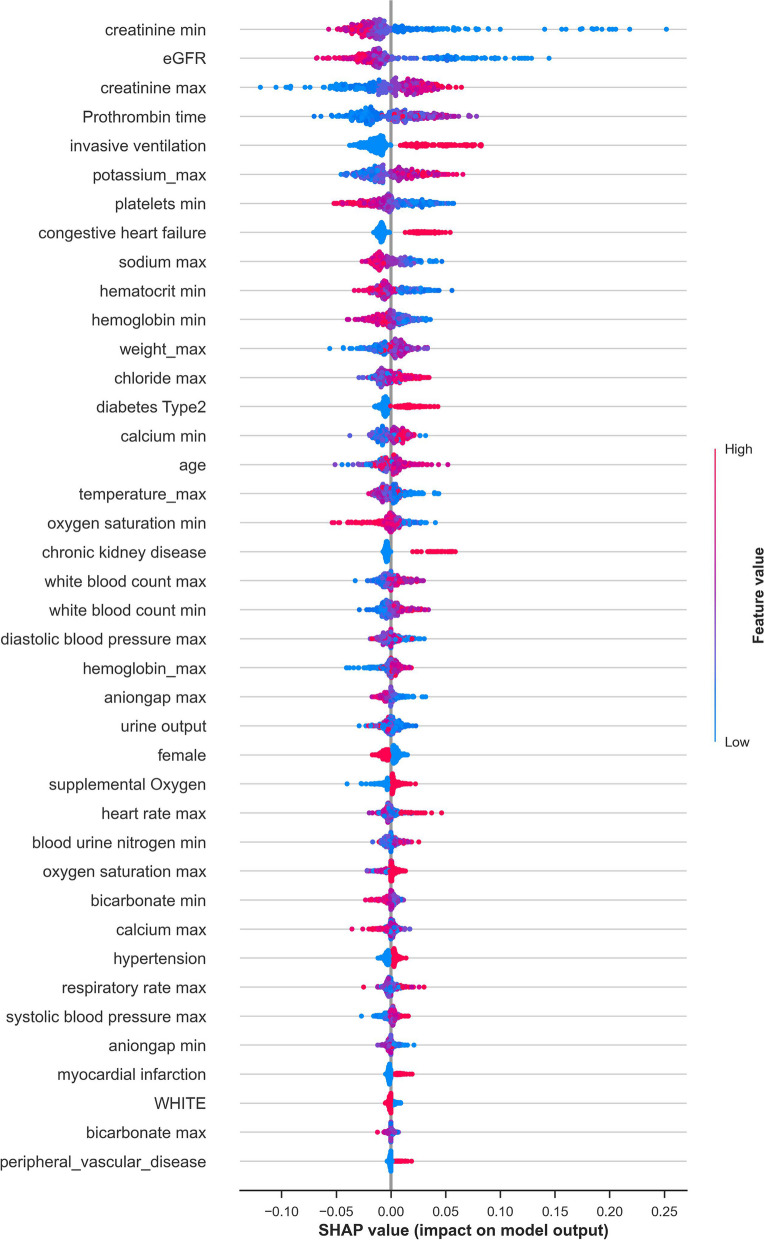
The feature importance of the XGB on the validation set using SHAP

To gain insights into the relative influence of each input variable and how it contributed to the model's output, the *summary_plot()* function in SHAP Python library [40] (Fig. 4) was used. The red and blue colours represent the relative influence of each input variable on the model's output, with red indicating a positive impact and blue indicating a negative impact. The colour intensity of each feature indicates the strength of its impact on the prediction outcome. The minimum sCr, prothrombin time, invasive ventilation, eGFR and maximum sCr were the key factors associated with AKI incidence. Hypercoagulability, hypoxemia, chronic kidney disease, diabetes Type 2 and age may also be the input features associated with the prediction of AKI incidence.

**Fig. 5:**
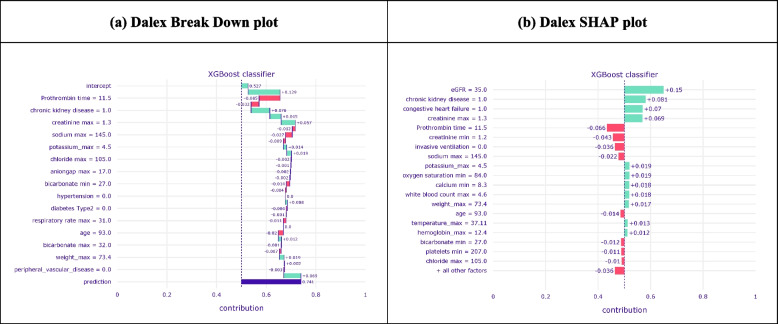
Local explanation of one single instance prediction with the Dalex break-down and SHAP plot

To explain model behaviour on the individual prediction level, we classified the prediction outcomes on the validation set into four categories: false positives (FP), true negatives (TN), false negatives (FN) and true positives (TP). Dalex python library [41] was used to explain instance-level on the FP group on a random selection of 10 predictions, visualised with a break-down (BD) plot. Because our focus was to explore the potential source of model failure while the KDIGO criteria had detected them as negative AKI cases. We randomly selected 10 prediction instances (see Supplementary Material 5) and observed that some of the FP predictions had relatively high creatinine, making them possible candidates for positive AKI cases; yet the KDIGO criteria classified them as negative cases. Figure (5-a) represents a break-down plot for a patient in the FP group with relatively high creatinine of 1.3 mg/dL and eGFR of 35.0 ml/min/1.73 m2. The green and red colours, in the BD plot, indicate the increase and decrease of prediction probability respectively for each variable for the occurrence of AKI. While the BD plot is an intuitive representation of variables’ contribution to the final prediction outcome, it only shows the additive attributions of each variable and rearranging the order of the variables may result in different feature importance representations.

To mitigate this limitation, we used the SHAP plot in the Dalex package which is based on averaging the value of a variable’s attribution over all (or many) possible orderings (Fig. 5-b). Similar to the BD plot, the SHAP plot indicated that maximum creatinine and eGFR during the first 24 h of admission are associated with an increased risk of AKI for this specific patient in the FP group.

Considering the sCr as a key predictor of AKI, we hypothesised that the magnitude of the difference between the baseline sCr and creatinine values on days 1 to 3 did not meet KDIGO criteria for the detection of AKI. Reviewing this patient’s actual baseline sCr (1.09 mg/dL) suggested that the elevated sCr (1.3 mg/dL) on the first day of admission did not satisfy the KDIGO criteria for an AKI episode. This analysis led us to conduct three experiments by modifying the previous three baseline sCr definitions, described earlier. Our motivation was to capture all possible baseline sCr measurements if existed prior to each admission. In our experiments, any unavailable pre-ICU sCr values were imputed using the MDRD backward equation.

- Baseline sCr 3 Modified. 1: The minimum of all pre-ICU sCr values in the 180 to 7 days before admission.
- Baseline sCr 3 Modified. 2: The minimum of all pre-ICU sCr values in the 180 to 0 days before admission.
- Baseline sCr 3 Modified. 3: The mean of all pre-ICU sCr values in the 180 to 0 days before admission.

The dataset was relabelled using the KDIGO definition with these three modified baselines and the same exclusion criteria were applied to each cohort. Three XGB models were trained on 60% of the data, calibrated on the validation set (20%) and tested on the unseen test data (20%). Hyperparameters tuning was performed with fivefold cross validation on each model to optimize the f1 score. Models were calibrated on the validation set and calibration metrics on the test sets. The discriminative performance of each calibrated model was assessed using the hold-out test (Fig. 2).

The patient cohort with the baseline sCr defined as mean 180 to 0 days prior to admission achieved a f1 score and precision, recall and AUC of 0.58, 0.54, 0.63 and 0.85 respectively. In this cohort, the calibration performance resulted in an ECE of 0.0726, a Brier score of 0.1000 and a calibration slope of 1.0970. The calibration performance of the models with the modified baselines was reported in Supplementary Material 6. This XGB's best hyperparameters were *model_n_estimators:* 200, *model_num_leaves*:10, *model_min_child_samples*:100, *model_min_child weight*: 1, *model_subsample*: 0.2, *model_reg_alpha*: 50 and *model_reg_lambda*: 0. Of all the 44,410 admissions in this cohort, 12,824 (28.87%) records with AKI on day 1 were excluded. The final cohort size was 31,586 admissions. The AKI cases were 5,473 (17.32%) which is slightly higher than the cohort with the 180 to 7 days pre-pre-ICU baseline sCr (16.70%). The characteristics of the candidate predictors used for training, validation and testing the XGB are provided in Table 1.

**Table 1:** Characteristics of candidate predictors for the best XGB after error analysis

Data source	Variable	Unit	Total data median (IQR)	Train set median (IQR)	Validation set Median (IQR)	Total data median (IQR)
Demographics	Age at admission	year	66.0 (23.0)	66.0 (23.0)	65.0 (22.0)	66.0 (23.0)
	Gender-N(%)	-				
	Female		17,608 (55.75%)	14,051 (55.61%)	2,874 (56.87%)	3,557 (56.3%)
	Male		13,978 (44.25%)	11,217 (44.39%)	2,180 (43.13%)	2,761 (43.7%)
	Ethnicity-N(%)	-				
	White		9,706 (30.73%)	7,779 (30.79%)	1,528 (30.23%)	1,927 (30.5%)
	African-American		2,432 (7.7%)	1,948 (7.71%)	360 (7.12%)	484 (7.66%)
	Hispanic-Latino		1,059 (3.35%)	829 (3.28%)	181 (3.58%)	230 (3.64%)
	Asian		943 (2.99%)	752 (2.98%)	147 (2.91%)	191 (3.02%)
	Other		5,272 (16.69%)	4,250 (16.82%)	840 (16.62%)	1,022 (16.18%)
Labs and vital signs	Prothrombin time	second	13.9 (3.6)	13.9 (3.7)	14.0 (3.7)	13.9 (3.5)
	Anion gap max	mEq/L	14.0 (5.0)	14.0 (5.0)	14.0 (5.0)	14.0 (5.0)
	Anion gap min	mEq/L	12.0 (4.0)	12.0 (4.0)	12.0 (4.0)	12.0 (4.0)
	Bicarbonate max	mEq/L	25.0 (4.0)	25.0 (4.0)	25.0 (4.0)	25.0 (4.0)
	Bicarbonate min	mEq/L	23.0 (4.0)	23.0 (4.0)	23.0 (4.0)	23.0 (4.0)
	Blood urine nitrogen min	mg/dL	14.0 (9.0)	14.0 (9.0)	14.0 (10.0)	14.0 (9.0)
	Calcium max	mg/dL	8.6 (0.9)	8.6 (0.9)	8.6 (0.9)	8.6 (0.9)
	Calcium min	mg/dL	8.3 (0.9)	8.3 (0.9)	8.3 (0.9)	8.3 (0.9)
	Chloride max	mEq/L	106.0 (6.0)	106.0 (6.0)	106.0 (6.0)	106.0 (6.0)
	Creatinine max	mg/dL	0.8 (0.3)	0.8 (0.3)	0.8 (0.3)	0.8 (0.2)
	Creatinine min	mg/dL	0.7 (0.3)	0.7 (0.3)	0.7 (0.3)	0.7 (0.3)
	Diastolic blood pressure max	mmHg	85.0 (23.0)	85.0 (24.0)	85.0 (23.0)	85.0 (23.0)
	eGFR	ml/min/1.73 m2	83.0 (34.0)	83.0 (34.0)	84.0 (33.0)	83.0 (33.0)
	Heart rate max	bpm	99.0 (25.0)	99.0 (25.0)	99.0 (24.25)	99.0 (25.0)
	Hematocrit min	%	31.4 (9.6)	31.4 (9.5)	31.4 (9.3)	31.5 (9.4)
	Hemoglobin min	g/dL	10.5 (3.2)	10.5 (3.2)	10.5 (3.0)	10.6 (3.2)
	Hemoglobin max	g/dL	11.7 (2.9)	11.7 (2.9)	11.7 (2.8)	11.8 (2.9)
	Oxygen saturation (Spo2) max	%	100.0 (1.0)	100.0 (1.0)	100.0 (1.0)	100.0 (1.0)
	Oxygen saturation (Spo2) min	%	93.0 (4.0)	93.0 (4.0)	93.0 (4.0)	93.0 (4.0)
	Platelets min	K/uL	180.0 (108.0)	180.0 (108.0)	179.0 (108.0)	179.0 (106.0)
	Potassium max	mEq/L	4.3 (0.6)	4.3 (0.6)	4.3 (0.6)	4.3 (0.6)
	Respiratory rate max	inspirations/min	26.0 (7.0)	26.0 (7.0)	26.0 (7.0)	26.0 (7.0)
	Sodium max	mEq/L	140.0 (5.0)	140.0 (5.0)	140.0 (5.0)	140.0 (5.0)
	Systolic blood pressure max	mmHg	146.0 (27.0)	146.0 (27.0)	146.0 (28.0)	146.0 (27.0)
	Temperature max	Celsius	37.22 (0.73)	37.22 (0.73)	37.22 (0.73)	37.22 (0.73)
	Urine output	ml	200.0 (250.0)	200.0 (250.0)	200.0 (250.0)	200.0 (250.0)
	Weight max	kg	80.0 (28.0)	79.8 (27.9)	79.6 (27.8)	80.1 (27.97)
	White blood count max	K/uL	12.1 (7.4)	12.1 (7.4)	12.1 (7.4)	12.1 (7.45)
	White blood count min	K/uL	9.2 (5.3)	9.2 (5.3)	9.2 (5.4)	9.2 (5.3)
Past medical history	Obesity -N(%)	-				
	No(0)		29,468 (93.29%)	23,558 (93.23%)	4,730 (93.59%)	5,910 (93.54%)
	Yes(1)		2,118 (6.71%)	1,710 (6.77%)	324 (6.41%)	408 (6.46%)
	Mild liver disease -N(%)	-				
	No(0)		28,979 (91.75%)	23,184 (91.75%)	4,624 (91.49%)	5,795 (91.72%)
	Yes(1)		2,607 (8.25%)	2,084 (8.25%)	430 (8.51%)	523 (8.28%)
	Supplemental Oxygen -N(%)	-				
	No(0)		18,110 (57.34%)	14,437 (57.14%)	2,895 (57.28%)	3,673 (58.14%)
	Yes(1)		13,476 (42.66%)	1,0831 (42.86%)	2,159 (42.72%)	2,645 (41.86%)
	Sepsis -N(%)	-				
	No(0)		28,767 (91.08%)	23,000 (91.02%)	4,596 (90.94%)	5,767 (91.28%)
	Yes(1)		2,819 (8.92%)	2,268 (8.98%)	458 (9.06%)	551 (8.72%)
	Peripheral vascular disease-N(%)	-				
	No(0)		28,351 (89.76%)	2,2679 (89.75%)	4,523(89.49%)	5,672 (89.78%)
	Yes(1)		3,235 (10.24%)	2,589 (10.25%)	531(10.51%)	646 (10.22%)
	Chronic heart failure-N(%)	-				
	No(0)		27,777 (87.94%)	2,2241 (88.02%)	4,436 (87.77%)	5,536 (87.62%)
	Yes(1)		3,809 (12.06%)	3,027 (11.98%)	618 (12.23%)	782 (12.38%)
	Chronic kidney disease-N(%)	-				
	No(0)		28,596 (90.53%)	22,830 (90.35%)	4,579 (90.6%)	5,766 (91.26%)
	Yes(1)		2,990 (9.47%)	2,438 (9.65%)	475 (9.4%)	552 (8.74%)
	Cyclosporine-N(%)	-				
	No(0)		31,566 (99.94%)	25,253 (99.94%)	5,053 (99.98%)	6,313 (99.92%)
	Yes(1)		20 (0.06%)	15 (0.06%)	1 (0.02%)	5 (0.08%)
	Hypertension-N(%)					
	No(0)	-	16,155 (51.15%)	12,934 (51.19%)	2,601(51.46%)	3,221 (50.98%)
	Yes(1)		15,431 (48.85%)	12,334 (48.81%)	2,453 (48.54%)	3,097 (49.02%)
	Severe liver disease-N(%)	-				
	No(0)		30,545 (96.7%)	24,420 (96.64%)	4,879 (96.54%)	6,125 (96.95%)
	Yes(1)		1,041 (3.3%)	848 (3.36%)	175 (3.46%)	193 (3.05%)
	Myocardial infarction-N(%)	-				
	No(0)		26,777 (84.77%)	21,432 (84.82%)	4,333 (85.73%)	5,345 (84.6%)
	Yes(1)		4,809 (15.23%)	3,836 (15.18%)	721 (14.27%)	973 (15.4%)
	Tracheostomy-N(%)	-				
	No(0)		31,499 (99.72%)	25,207 (99.76%)	5,044 (99.8%)	6,292 (99.59%)
	Yes(1)		87 (0.28%)	61 (0.24%)	10 (0.2%)	26 (0.41%)
	Congestive heart failure-N(%)	-				
	No(0)		25,137 (79.58%)	20,139 (79.7%)	4,042 (79.98%)	4,998 (79.11%)
	Yes(1)		6,449 (20.42%)	5,129 (20.3%)	1,012 (20.02%)	1,320 (20.89%)
	Invasive ventilation-N(%)	-				
	No(0)		22,236 (70.4%)	17,755 (70.27%)	3,544 (70.12%)	4,481 (70.92%)
	Yes(1)		9,350 (29.6%)	7,513 (29.73%)	1,510 (29.88%)	1,837 (29.08%)
	Diabetes Type2-N(%)	-				
	No(0)		23,896 (75.65%)	19,140 (75.75%)	3,893 (77.02)	4,756 (75.28%)
	Yes(1)		7,690 (24.35%)	6,128 (24.25%)	1,161 (22.97%)	1,562 (24.72%)
	Chronic pulmonary disease-N(%)	-				
	No(0)		24,177 (76.54%)	19,321 (76.46%)	3,861 (76.39%)	4,856 (76.86%)
	Yes(1)		7,409 (23.46%)	5,947 (23.54%)	1,193 (23.61%)	1,462 (23.14%)
Outcome	AKI cases – N(%)	-	5,473 (17.32%)	3,502 (17.32%)	876 (17.33%)	1,095 (17.33%)

*Abbreviation*: *IQR* Interquartile range

## Discussion

In this study, we performed our analysis with the MIMIC-IV data and the KDIGO definition to identify AKI events with three different methods of estimating baseline sCr. ML models were developed for each cohort and the efficacy of the models was assessed. Using input data from the first 24 h of ICU admission, the goal of the ML models was to predict AKI on days 2 and 3 of ICU admission. We conducted model calibration in this study and reported the performance metrics of the calibrated models. Calibration is a vital step to determine the effectiveness of models which was rarely assessed in previous AKI prediction models [18]. We performed statistical comparisons to assess the significance of the variations in XGB models across all baselines. To the best of our knowledge, this is the first study that developed a suite of ML models for the prediction of AKI with different methods of baseline sCr. Overall, the results indicated that the selection of different baseline sCr methods may impact the performance of ML models for the prediction of AKI incidence. The XGB model yielded an AUC of 0.76, recall of 0.53, precision of 0.37, and f1 score of 0.43 for the baseline defined as the mean of sCr 180 to 7 days prior to ICU admission. We also analysed the model errors using XAI techniques. As a result, we relabelled the data with a new baseline method defined as the mean of sCr values in 180 to 0 days pre-admissions and achieved an AUC of 0.85, recall of 0.63, precision of 0.54 and f1 score of 0.58. This attempt allowed us to gain insight into the underlying causes of FP events in our ML models. Such understanding has the potential to enhance patient safety and mitigate alert fatigue by preventing unnecessary interventions when deploying ML analytics in real-world clinical settings [42]. Further observational and interventional clinical trials are essential to validate the findings of this study prior to considering any immediate clinical applications.

Currently, the KDIGO definition is the most widely accepted and used in the kidney community; however, there are limitations to utilising this definition for the diagnosis of AKI. The KDIGO definition largely relies on changes in sCr measurements and urine output which lack the required kinetic characteristics for real-time evaluation of kidney function in acute settings, especially when renal function varies abruptly [43]. Furthermore, both sCr measurements and urine output are commonly used to assess kidney function, but they are not specific to kidney diseases and can be impacted by other factors such as dehydration, diet, certain medication or liver disease [44]. More importantly, the KDIGO definition depends on the value of baseline sCr while there is no standard method of estimating baseline sCr [45]. Inaccurate baseline sCr estimation may result in the misclassification of AKI events and compromises the accuracy and comparability in the studies of AKI. Based on the target population, the clinical setting and the availability of inpatients or outpatient baseline sCr, it is vital to establish a standardised definition of baseline sCr to facilitate comparability in future AKI research. Furthermore, employing a standardised baseline definition that accounts for the variability of diverse demographics and regions, can lead to fair and robust external validations to establish the reliability and generalisability of ML-based products, making them more trustworthy and suitable for integration into the clinical workflow.

ML models can be developed with two software development architectures: model-driven architecture (MDA) and data-driven architecture (DDA). Based on the MDA paradigm, model development relies on prior knowledge to guide the design and development and can be useful in situations where the problem domain is well-understood and the existing knowledge can guide the development process [46]. This can include the selection of model algorithms, hyperparameters and input features. In contrast, the DDA emphasises the exploration and analysis of available data to guide the design, development, and optimization of models when the problem domain is complex or poorly understood [47]. The DDA involves using statistical and computational techniques to obtain meaningful patterns and insights from complex datasets and using these findings to inform the selection of appropriate ML algorithms, features, and hyperparameters. Although the MDA influenced the ML development methodologies of most studies related to the prediction of AKI [17, 18], a combination of both architectures guided the project design and development of this study. The input features were identified based on literature review and consultation with kidney experts in our research team to guide the model developments. We then used hyperparameter tuning to find the optimal set of input features for performance optimisation. We adopted Explainable AI (XAI) techniques to understand the underlying factors associated with the model errors. XAI techniques can assist end-users to uncover errors in model output, enabling them to improve the model's performance and avoid potentially harmful decisions or actions in future implementations. With the increasing development of AI-based models in healthcare, it is crucial to ensure that these models are interpretable and explainable to facilitate the integration of ML-based models in routine clinical care.

There are some limitations to take into account when considering the findings of this study. The first limitation is related to the cohort selection process in this study. Only patients who had at least one sCr test on days 1, 2, and 3 following admissions were included. This selection criterion was necessary because the detection of AKI relies on sCr measurements on each of these days. However, this approach may introduce some selection bias as it excludes patients who did not have sCr measurements during this specific timeframe. The second limitation is the exclusion of patient records with AKI on day 1 as our focus was specifically on detecting iatrogenic cases of AKI. This can also introduce selection bias as there may be dissimilarities between patients who have sufficient data for evaluating AKI on day 1 and those who were retained in the study. It is important to consider these limitations when generalising the findings to a broader population. Third, we attempted to mitigate the impact of data missingness by employing an imputation technique. However, it is worth noting that this approach may introduce potential assumptions and biases that should be considered when assessing the findings. Nonetheless, the availability of baseline sCr is not random and can be influenced by various factors such as variations in data collection practices, differences in patient history documentation and the availability of previous laboratory test results. Fourth, our analysis was confined to the ICU in a specific health system. The mix of cases in the ICU can vary across countries, leading to different levels of data completeness for various reasons. Additionally, we recognise that AKI cases in the ICU may not be representative of the majority of AKI cases occurring in non-intensive care settings. The patients in the ICU often have more severe medical conditions, which can result in different patterns and characteristics compared to the broader AKI population. These limitations can affect the generalisability of our findings to a broader population of AKI cases outside of the ICU. Fifth, we cannot be certain that the estimated baseline creatine using the MDRD backwards calculation reflects the real stable baseline kidney function for those patients who had missing outpatient sCr measurements. Finally, the findings of the present study were based on a small variation of baseline methods, limiting their generalisability to a broader spectrum of baselines. Before any clinical applications, future research should be conducted to rigorously validate the findings of this study across a more diverse array of baseline variations.

## Conclusion

In conclusion, varying baseline sCr methods may impact the performance of ML models in predicting AKI incidence. To minimise performance variations caused by different baseline methods and improve the consistency of AKI prediction, a standard method of baseline sCr is needed that is clinically relevant and widely applicable to facilitate the effective use of AI in AKI prediction and management. In healthcare, XAI techniques can help AI developers and end users to better understand how AI models are making predictions, which can increase trust and confidence in the technology. XAI techniques can also help identify errors in the data used to train the AI models, enabling clinicians to make informed decisions and improve the overall quality of care.

### Supplementary Information

**Additional file 1: Supplementary material 1.** TRIPOD Checklist: Prediction Model Development and Validation. **Supplementary Material 2.** Candidate predictors of this study for ML development. **Supplementary Material 3.** The missingness counts of the input variables (*n*=*****). **Supplementary Material 4.** The performance of ML models along with the selected classification thresholds on the validation sets. **Supplementary Material 5.** The Break Down (BD) and SHAP plots using the Dalex package for 10 random prediction instances in the false positive group. **Supplementary Material 6.** Calibration curves of three modified baselines.

## Data Availability

The datasets generated and/or analysed during the current study are available in the MIMIC-IV v1.0 repository, https://doi.org/10.13026/s6n6-xd98. The underlying code for this study is not publicly available but may be made available to qualified researchers on reasonable request from the corresponding author.
