# Supplementary material for: Machine learning clinical prediction models for acute kidney injury: the impact of baseline creatinine on prediction efficacy
Source: BMC Med Inform Decis Mak. 2023 Oct 9;23:207. doi: 10.1186/s12911-023-02306-0 (PMC10563357; doi:10.1186/s12911-023-02306-0)
Supplement: Supplementary file 1 — Additional file 1: Supplementary material 1. TRIPOD Checklist: Prediction Model Development and Validation. Supplementary Material 2. Candidate predictors of this study for ML development. Supplementary Material 3. The missingness counts of the input variables (n=*****). Supplementary Material 4. The performance of ML models along with the selected classification thresholds on the validation sets. Supplementary Material 5. The Break Down (BD) and SHAP plots using the Dalex package for 10 random prediction instances in the false positive group. Supplementary Material 6. Calibration curves of three modified baselines. [file 12911_2023_2306_MOESM1_ESM.docx]

## Supplementary Materials

Supplementary material 1: TRIPOD Checklist: Prediction Model Development and Validation

| **Section/Topic** | **Item** |  | **Checklist Item** | **Page** |
| --- | --- | --- | --- | --- |
| **Title and abstract** | | | | |
| Title | 1 | D;V | Identify the study as developing and/or validating a multivariable prediction model, the target population, and the outcome to be predicted. | 1 |
| Abstract | 2 | D;V | Provide a summary of objectives, study design, setting, participants, sample size, predictors, outcome, statistical analysis, results, and conclusions. | 2 |
| **Introduction** | | | | |
| Background and objectives | 3a | D;V | Explain the medical context (including whether diagnostic or prognostic) and rationale for developing or validating the multivariable prediction model, including references to existing models. | 3 |
|  | 3b | D;V | Specify the objectives, including whether the study describes the development or validation of the model or both. | 4 |
| **Methods** | | | | |
| Source of data | 4a | D;V | Describe the study design or source of data (e.g., randomized trial, cohort, or registry data), separately for the development and validation data sets, if applicable. | 4 |
|  | 4b | D;V | Specify the key study dates, including start of accrual; end of accrual; and, if applicable, end of follow-up. | 4 |
| Participants | 5a | D;V | Specify key elements of the study setting (e.g., primary care, secondary care, general population) including number and location of centres. | 4 |
|  | 5b | D;V | Describe eligibility criteria for participants. | 5 |
|  | 5c | D;V | Give details of treatments received, if relevant. | N/A |
| Outcome | 6a | D;V | Clearly define the outcome that is predicted by the prediction model, including how and when assessed. | 5 |
|  | 6b | D;V | Report any actions to blind assessment of the outcome to be predicted. | N/A |
| Predictors | 7a | D;V | Clearly define all predictors used in developing or validating the multivariable prediction model, including how and when they were measured. | 5 |
|  | 7b | D;V | Report any actions to blind assessment of predictors for the outcome and other predictors. | N/A |
| Sample size | 8 | D;V | Explain how the study size was arrived at. | 9 |
| Missing data | 9 | D;V | Describe how missing data were handled (e.g., complete-case analysis, single imputation, multiple imputation) with details of any imputation method. | 6 |
| Statistical analysis methods | 10a | D | Describe how predictors were handled in the analyses. | 6 |
|  | 10b | D | Specify type of model, all model-building procedures (including any predictor selection), and method for internal validation. | 6 |
|  | 10c | V | For validation, describe how the predictions were calculated. | 6 |
|  | 10d | D;V | Specify all measures used to assess model performance and, if relevant, to compare multiple models. | 6 |
|  | 10e | V | Describe any model updating (e.g., recalibration) arising from the validation, if done. | 7 |
| Risk groups | 11 | D;V | Provide details on how risk groups were created, if done. | N/A |
| Development vs. validation | 12 | V | For validation, identify any differences from the development data in setting, eligibility criteria, outcome, and predictors. | 12,13 |
| **Results** | | | | |
| Participants | 13a | D;V | Describe the flow of participants through the study, including the number of participants with and without the outcome and, if applicable, a summary of the follow-up time. A diagram may be helpful. | 8 |
|  | 13b | D;V | Describe the characteristics of the participants (basic demographics, clinical features, available predictors), including the number of participants with missing data for predictors and outcome. | 13 |
|  | 13c | V | For validation, show a comparison with the development data of the distribution of important variables (demographics, predictors and outcome). | 13 |
| Model development | 14a | D | Specify the number of participants and outcome events in each analysis. | 8 |
|  | 14b | D | If done, report the unadjusted association between each candidate predictor and outcome. | N/A |
| Model specification | 15a | D | Present the full prediction model to allow predictions for individuals (i.e., all regression coefficients, and model intercept or baseline survival at a given time point). | 13 |
|  | 15b | D | Explain how to the use the prediction model. | 5 |
| Model performance | 16 | D;V | Report performance measures (with CIs) for the prediction model. | 7 |
| Model-updating | 17 | V | If done, report the results from any model updating (i.e., model specification, model performance). | 8 |
| **Discussion** | | | | |
| Limitations | 18 | D;V | Discuss any limitations of the study (such as nonrepresentative sample, few events per predictor, missing data). | 16 |
| Interpretation | 19a | V | For validation, discuss the results with reference to performance in the development data, and any other validation data. | 15 |
|  | 19b | D;V | Give an overall interpretation of the results, considering objectives, limitations, results from similar studies, and other relevant evidence. | 17 |
| Implications | 20 | D;V | Discuss the potential clinical use of the model and implications for future research. | 17 |
| **Other information** | | | | |
| Supplementary information | 21 | D;V | Provide information about the availability of supplementary resources, such as study protocol, Web calculator, and data sets. | included |
| Funding | 22 | D;V | Give the source of funding and the role of the funders for the present study. | 17 |

D: development of a prediction model, V: validation of a prediction model, D;V: development and validation of a prediction model, N/A: Not Applicable

Supplementary Material 2: Candidate predictors of this study for ML development

| **Data source** | **Variable** | **Unit** | **ICD codes 9-10** |
| --- | --- | --- | --- |
| Demographics | Age at admission | year |  |
|  | Gender-N(%)  Female  Male | - | - |
|  | Ethnicity-N(%)  White  African-American  Hispanic-Latino  Asian  Other | - | - |
| Labs | Prothrombin time | second | - |
|  | Anion gap max | mEq/L | - |
|  | Anion gap min | mEq/L | - |
|  | Bicarbonate max | mEq/L | - |
|  | Bicarbonate min | mEq/L | - |
|  | Blood urine nitrogen min | mg/dL | - |
|  | Calcium max | mg/dL | - |
|  | Calcium min | mg/dL | - |
|  | Chloride max | mEq/L | - |
|  | Creatinine max | mg/dL | - |
|  | Creatinine min | mg/dL | - |
|  | Diastolic blood pressure max | mmHg | - |
|  | eGFR | ml/min/1.73 m2 | - |
|  | Heart rate max | bpm | - |
|  | Hematocrit min | % | - |
|  | Hemoglobin min | g/dL | - |
|  | Hemoglobin max | g/dL | - |
|  | Oxygen saturation (Spo2) max | % | - |
|  | Oxygen saturation (Spo2) min | % | - |
|  | Platelets min | K/uL | - |
|  | Potassium max | mEq/L | - |
|  | Respiratory rate max | inspirations/min | - |
|  | Sodium max | mEq/L | - |
|  | Systolic blood pressure max | mmHg | - |
|  | Temperature max | Celsius | - |
|  | Urine output | ml | - |
|  | Weight max | kg | - |
|  | White blood count max | K/uL | - |
|  | White blood count min | K/uL | - |
| Comobidities | Obesity | - | ICD9:27801,V778  ICD10: E66 |
|  | Mild liver disease | - | ICD9:570,571,0706,0709,5733,5734,5738,5739,V427,07022,07023,07032,07033,07044,07054 |
|  |  |  | ICD10:B18,K73,K74,K700,K701,K702,K703,K709,K713,K714,K715,K717,K760,K762,K763,K764,K768,K769,Z944 |
|  | Severe liver disease | - | ICD9: 4560,4561,4562,5722, 5723, 5724, 5725, 5726, 5727,5728 |
|  |  |  | ICD10:I850,I859,I864,I982,K704,K711,K721,K729,K765,K766,K767 |
|  | Supplemental Oxygen | - | - |
|  | Sepsis | - | ICD9: 99591,99592 |
|  |  |  | ICD10: A41,R652 |
|  | Peripheral vascular disease | - | ICD9:440,441,0930,4373,4471,5571,5579,V434,4431, 4431, 4432, 4433, 4434, 4435, 4436,4437, 4438, 4439 |
|  |  |  | ICD10:I70,I71,I731,I738,I739,I771,I790,I792,K551,K558,K559,Z958,Z959 |
|  | Chronic heart failure | - | ICD9:40401,40403,40411,40413,40491,40493,42820,42822,42830,42832,42840,42842 4249,4281 |
|  |  |  | ICD10:I50,I509,I502,I503,I504,I508,I5020,I5030,I5032,I5040,I5042,I5081,I5082,I5083,I5084,I5089,I50810,I50812,I50814 |
|  | Chronic kidney disease | - | ICD9:582,585,586,V56,5880,V420,V451,40301,40311,40391,40402,40403,40412,40413,40492,40493 |
|  |  |  | ICD10:N18,N19,I120,I131,N032,N033,N034,N035,N036,N037,N052,N053,N054,N055,N056,N057,N250,Z490,Z491,Z492,Z940,Z992 |
|  | kidney transplant | - | ICD9: V45, V4511, V420 |
|  |  |  | ICD10: Z992, Z94 |
|  | Cyclosporine | - | - |
|  | Hypertension | - | ICD9:4010,4011,4019,2507,40501,40509,40511,40519,40591,40599,64200,64201,64202,64203,64204,64220,64221,64222,64223,64224,64270,64271,64272,64273,64274 |
|  |  |  | ICD10:I10,I15,O10,O11,O12,I150,I158,I159,O100,O104,O109 |
|  | Myocardial infarction | - | ICD9:410,412 |
|  |  |  | ICD10:I21,I22,I252 |
|  | Tracheostomy | - | - |
|  | Congestive heart failure | - | ICD9:428,39891,40201,40211,40291,40401,40403,40411,40413,40491,40493,4254,4259 |
|  |  |  | ICD10:I43,I50,I099,I110,I130,I132,I255,I420,I425,I426,I427,I428,I429,P290 |
|  | Invasive ventilation | - | - |
|  | Diabetes Type2 | - | ICD9: 2500,2501,2502,2503,2508,2509, 2504,2505,2506,2507 |
|  |  |  | ICD10:E100,E10l,E106,E108,E109,E110,E111,E116,E118,E119,E120,E121,E126,E128,E129,E130,E131,E136,E138,E139,E140,E141,E146,E148,E149,E102,E103,E104,E105,E107,E112,E113,E114,E115,E117,E122,E123,E124,E125,E127,E132,E133,E134,E135,E137,E142,E143,E144,E145,E147 |
|  | Chronic pulmonary disease | - | ICD9:490,505,4168,4169,5064,5081,5088 |
|  |  |  | ICD10:J40, J41, J42, J43, J44, J45, J46, J47,J60, J61, J62, J63, J64, J65, J66, J67, I278,I279,J684,J701,J703 |

Supplementary Material 3: The missingness counts of the input variables (n=*****)

| **Variable** | | **No of Missingness(%)** |
| --- | --- | --- |
| Age | | 0(0.0) |
| Female | | 0(0.0) |
| Ethnicity | | 7,520(16.93) |
| Chronic kidney disease | | 0(0.0) |
| Kidney transplant | | 0(0.0) |
| Congestive heart failure | | 0(0.0) |
| Diabetes Type2 | | 0(0.0) |
| Hypertension | | 0(0.0) |
| Obesity | | 0(0.0) |
| Peripheral vascular disease | | 0(0.0) |
| Mild liver diseases | | 0(0.0) |
| Severe liver diseases | | 0(0.0) |
| Myocardial infarction | | 0(0.0) |
| Chronic pulmonary disease | | 0(0.0) |
| Chronic heart failure | | 0(0.0) |
| Sepsis |  | 0(0.0) |
| Hematocrit min | | 63(0.14) |
| Hemoglobin min | | 82(0.18) |
| Hemoglobin max | | 82(0.18) |
| Platelets min | | 876(1.97) |
| White blood count min | | 268(0.6) |
| White blood count max | | 268(0.6) |
| Albumin min | | 27,699(62.37) |
| Albumin max | | 27,699(62.37) |
| Globulin min | | 43,829(98.69) |
| Globulin max | | 43,829(98.69) |
| Total protein min | | 43,401(97.73) |
| Total protein max | | 43,401(97.73) |
| Anion gap min | | 105(0.24) |
| Anion gap max | | 105(0.24) |
| Bicarbonate min | | 53(0.12) |
| Bicarbonate max | | 53(0.12) |
| Blood urine nitrogen min | | 30(0.07) |
| Calcium min | | 4,066(9.16) |
| Calcium max | | 4,066(9.16) |
| Chloride max | | 52(0.12) |
| Creatinine min | | 0(0.0) |
| Creatinine max | | 0(0.0) |
| Sodium max | | 57(0.13) |
| Potassium max | | 86(0.19) |
| Prothrombin time | | 3,869(8.71) |
| Thrombin min | | 44,333(99.83) |
| Thrombin max | | 44,333(99.83) |
| Bilirubin min | | 22,581(50.85) |
| Bilirubin max | | 22,581(50.85) |
| eGFR | | 0(0.0) |
| Heart rate max | | 78(0.18) |
| Systolic blood pressure max | | 198(0.45) |
| Diastolic blood pressure max | | 199(0.45) |
| Respiratory rate max | | 115(0.26) |
| Temperature max | | 1,468(3.31) |
| Oxygen saturation min | | 89(0.2) |
| Oxygen saturation max | | 89(0.2) |
| Cyclosporine | | 0(0.0) |
| BMI | | 21,352(48.08) |
| Urine output | | 641(1.44) |
| Supplemental Oxygen | | 0(0.0) |
| Invasive ventilation | | 0(0.0) |
| Tracheostomy | | 0(0.0) |
| Weight max | | 438(0.99) |

Supplementary Material 4: The performance of ML models along with the selected classification thresholds on the validation sets

| **Selected baseline SCr** | **Model** | **Classification**  **Threshold** | **Precision** | **Recall** | **f1** | **AUC** |
| --- | --- | --- | --- | --- | --- | --- |
| Baseline SCr 1 | LR | 0.26 | 0.43 | 0.52 | 0.47 | 0.74 |
|  | ANN | 0.26 | 0.41 | 0.56 | 0.47 | 0.73 |
|  | RF | 0.26 | 0.39 | 0.54 | 0.45 | 0.72 |
|  | XGB | 0.23 | 0.43 | 0.51 | 0.46 | 0.75 |
| Baseline SCr 2 | LR | 0.26 | 0.36 | 0.55 | 0.44 | 0.73 |
|  | ANN | 0.27 | 0.46 | 0.42 | 0.42 | 0.73 |
|  | RF | 0.24 | 0.37 | 0.49 | 0.42 | 0.72 |
|  | XGB | 0.23 | 0.38 | 0.50 | 0.43 | 0.73 |
| Baseline SCr 3 | LR | 0.23 | 0.37 | 0.46 | 0.41 | 0.74 |
|  | ANN | 0.21 | 0.35 | 0.50 | 0.42 | 0.74 |
|  | RF | 0.25 | 0.36 | 0.55 | 0.43 | 0.74 |
|  | XGB | 0.15 | 0.37 | 0.53 | 0.43 | 0.77 |
| Baseline SCr 3 mod. 1 | XGB | 0.21 | 0.36 | 0.65 | 0.47 | 0.78 |
| Baseline SCr 3 mod. 2 | XGB | 0.26 | 0.46 | 0.47 | 0.46 | 0.77 |
| Baseline SCr 3 mod. 3 | XGB | 0.25 | 0.54 | 0.63 | 0.58 | 0.85 |

ANN: Artificial Neural Networks, LR: Logistic Regression, RF: Random*, RRT: Renal Replacement Therapy,* Forest, SCr: Serum Creatinine, XGB: Extreme Gradient Boosting

Supplementary Material 5: The Break Down (BD) and SHAP plots using the Dalex package for 10 random prediction instances in the false positive group

| **Row#** | **sCr max (mg/dL)** | **Baseline sCr (mg/dL)** | **(a) BD plot** | **(b) SHAP plot** |
| --- | --- | --- | --- | --- |
| **1** | 1.1 | 1.04 | 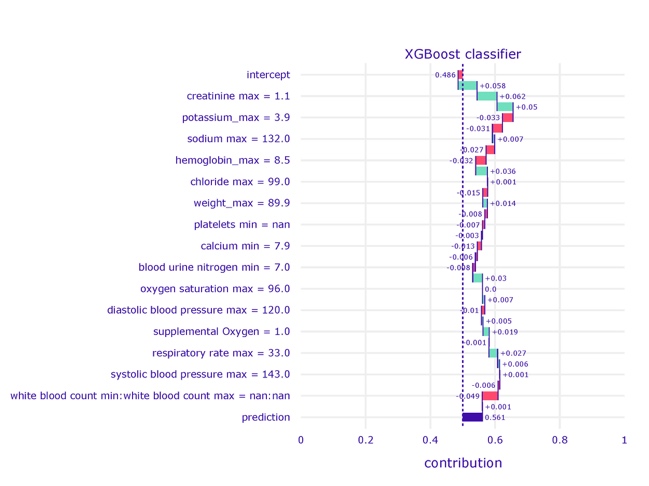  Figure S1 (1-a). BD plot for the patient #1 in FP group | 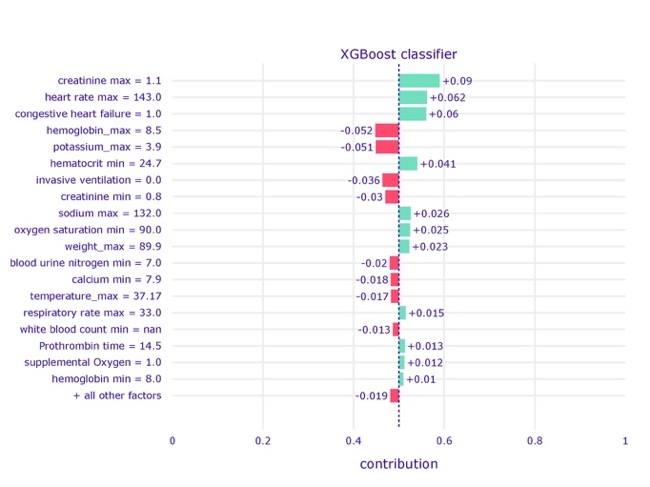  Figure S1 (1-b). SHAP plot for the patient #1 in FP group |
| **2** | 1.3 | 1.04 | 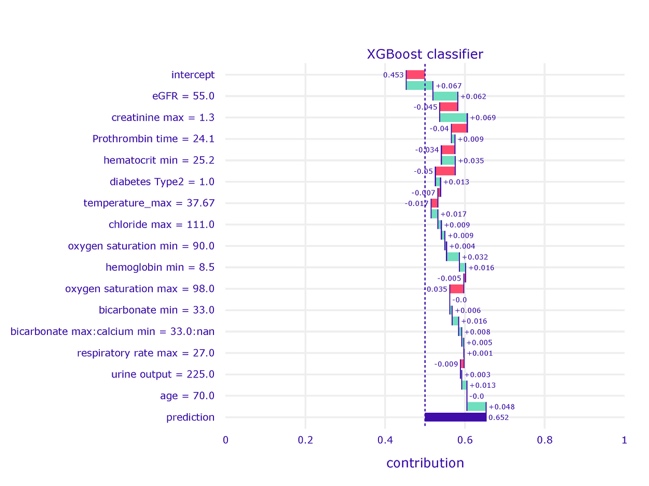  Figure S1 (2-a). BD plot for the patient #2 in FP group | 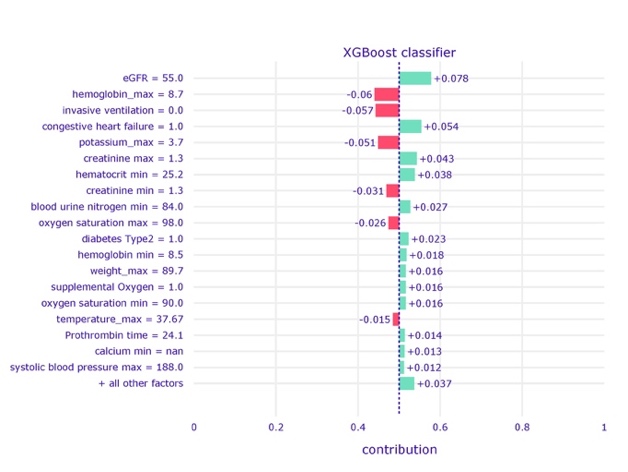  Figure S1 (2-b). SHAP plot for the patient #2 in FP group |
| **3** | 1.2 | 1.1 | 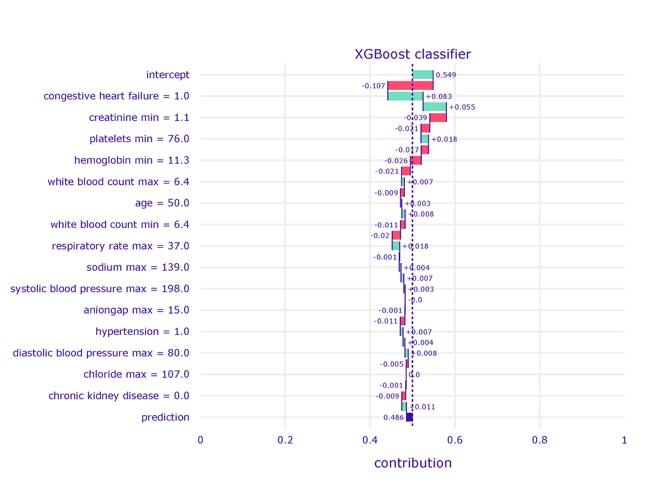  Figure S1 (3-a). BD plot for the patient #3 in FP group | 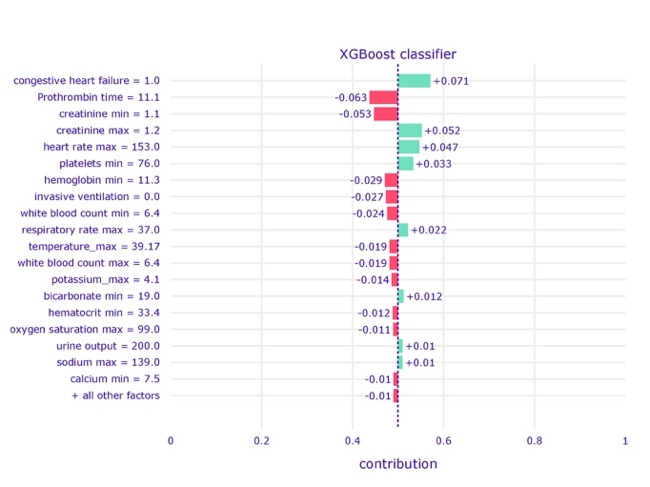  Figure S1 (3-b). SHAP plot for the patient #3 in FP group |
| **4** | 2.4 | 2.41 | 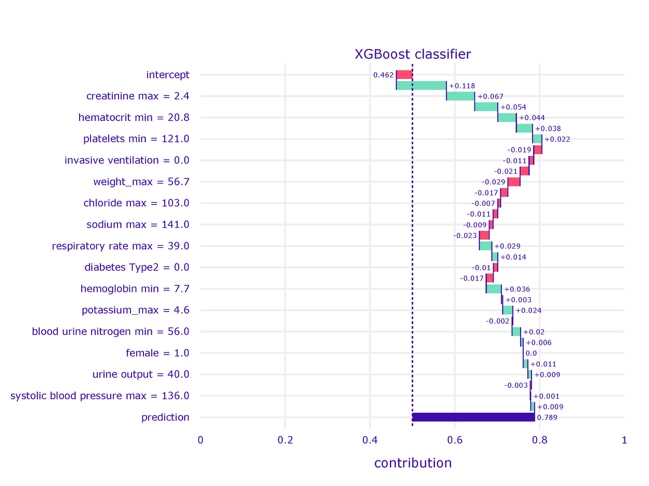  Figure S1 (4-a). BD plot for the patient #4 in FP group | 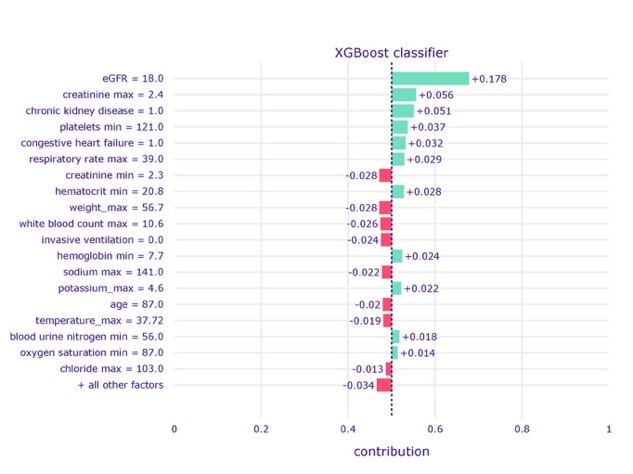  Figure S1 (4-b). SHAP plot for the patient #4 in FP group |
| **5** | 1.5 | 1.49 | 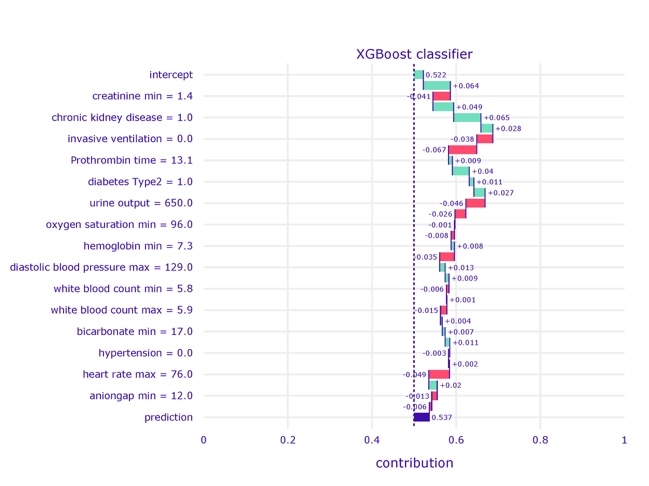  Figure S1 (5-a). BD plot for the patient #5 in FP group | 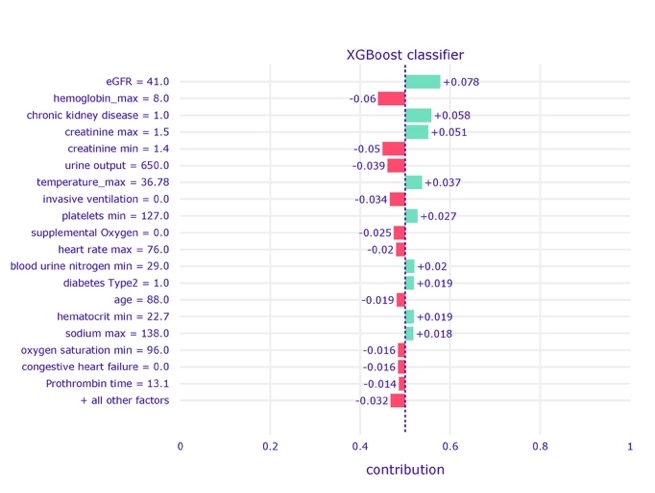  Figure S1 (5-b). SHAP plot for the patient #5 in FP group |
| **6** | 1.1 | 1.01 | 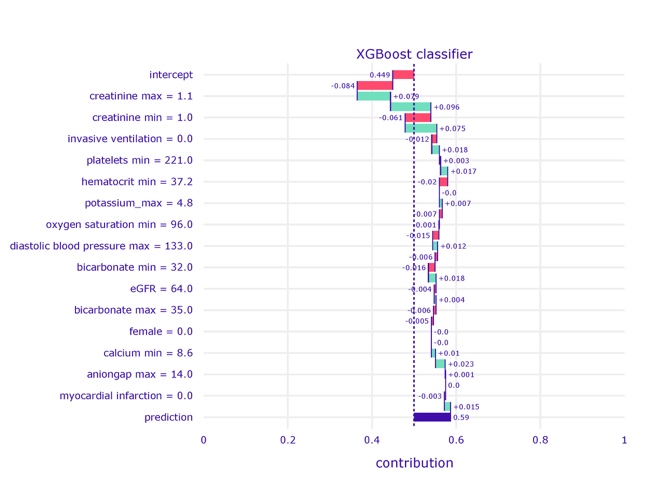  Figure S1 (6-a). BD plot for the patient #6 in FP group | 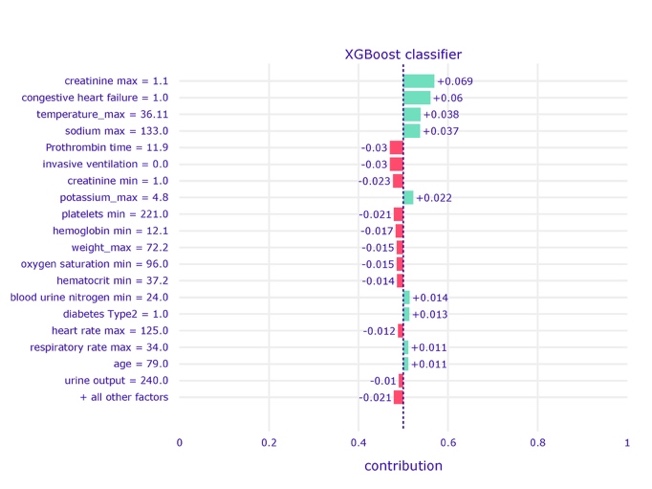  Figure S1 (6-b). SHAP plot for the patient #6 in FP group |
| **7** | 1.2 | 1.04 | 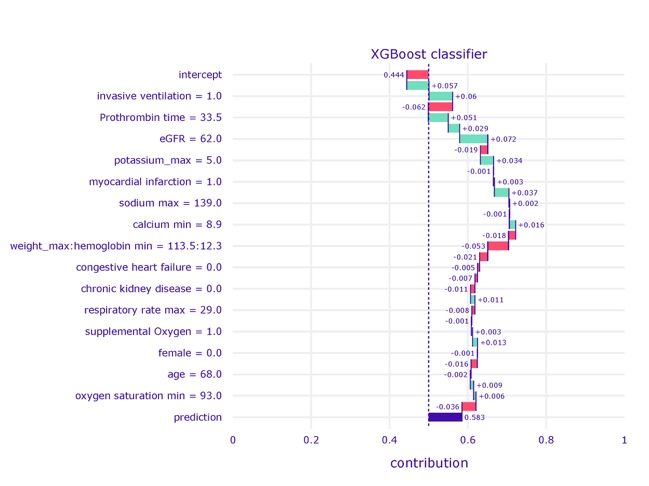  Figure S1 (7-a). BD plot for the patient #7 in FP group | 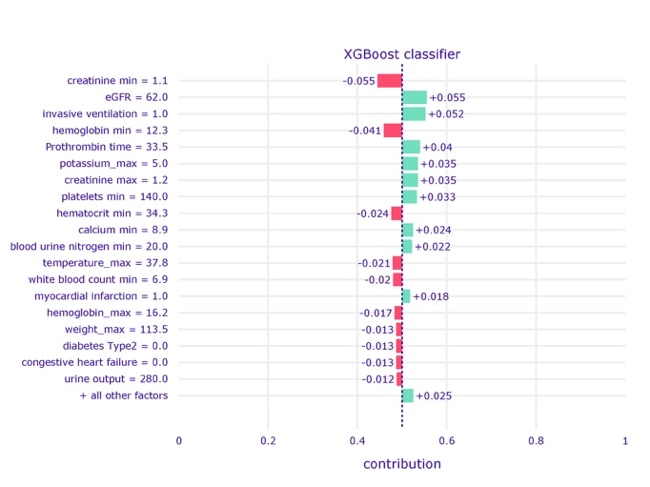  Figure S1 (7-b). SHAP plot for the patient #7 in FP group |
| **8** | 1.0 | 1.01 | 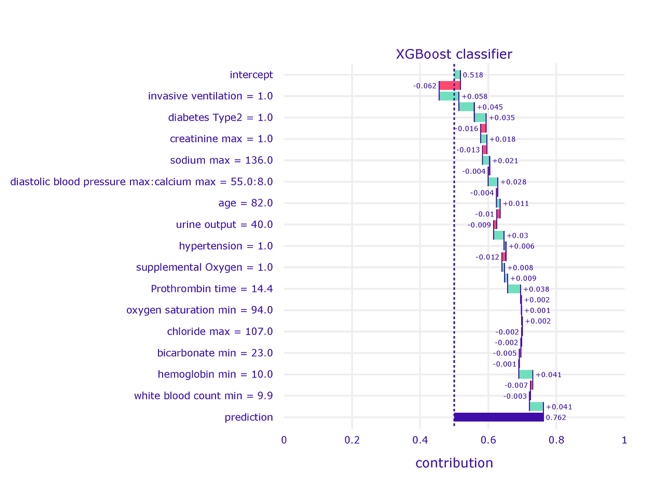  Figure S1 (8-a). BD plot for the patient #8 in FP group | 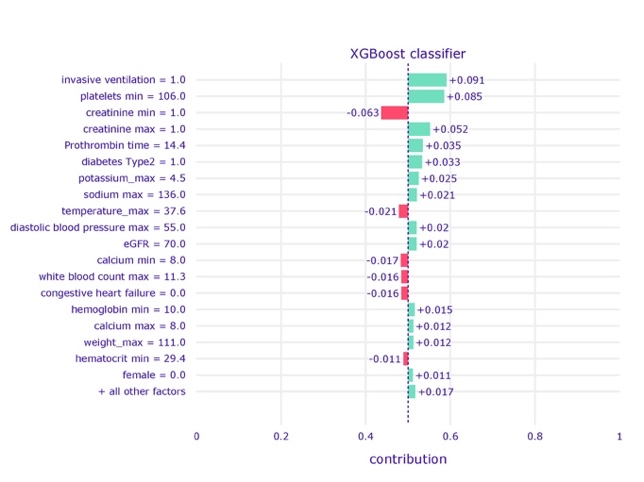  Figure S1 (8-b). SHAP plot for the patient #8 in FP group |
| **9** | 1.1 | 1.02 | 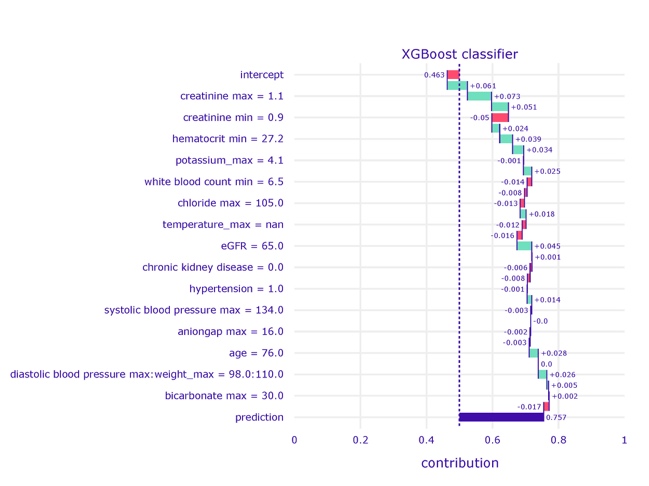  Figure S1 (9-a). BD plot for the patient #9 in FP group | 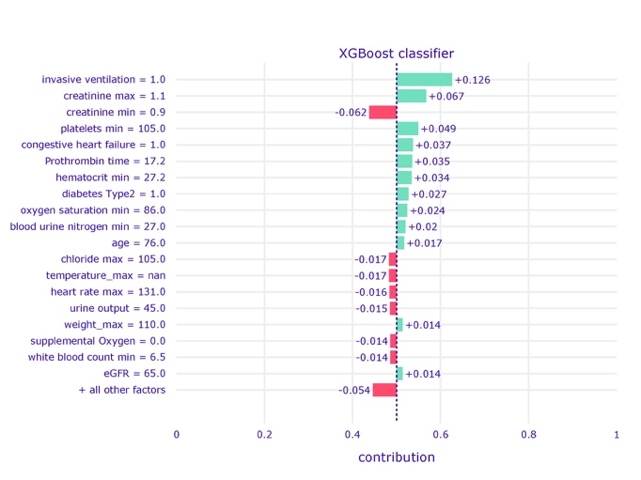  Figure S1 (9-b). SHAP plot for the patient #9 in FP group |
| **10** | 1.1 | 1.04 | 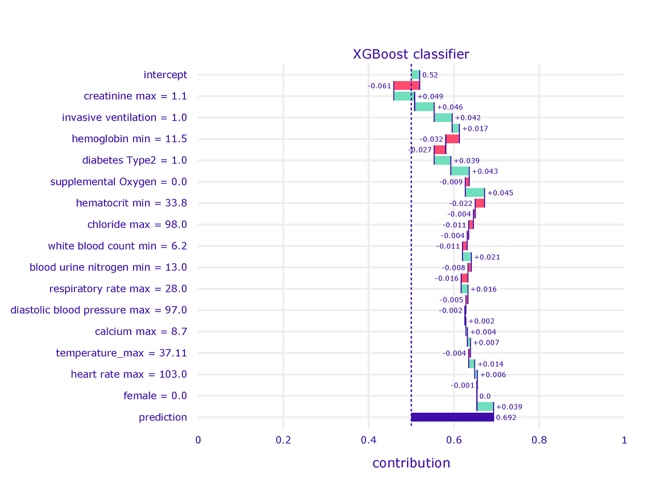  Figure S1 (10-a). BD plot for the patient #10 in FP group | 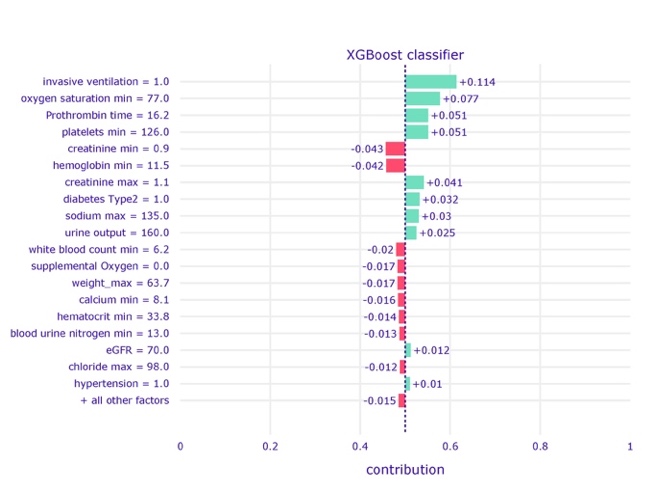  Figure S1 (10-b). SHAP plot for the patient #10 in FP group |

*Abbreviation: BD, Break Down;FP, False Positive; sCr, serume creatinine*

*Legend*: (a) DALEX Break Down (BD) plots display the impact of individual features on the false positive predictions of eXtreme Gradient Boosting (XGB) model. The x-axis represents the contribution of each input features to the model output. The y-axis lists the features being analysed, indicating the factors under consideration. Each bar corresponds to a specific feature's contribution. Green bars indicate a higher positive impact, while red bars signify a reduction in the model's output. (b) SHAP plots illustrate the aggregated contribution of individual input features to the output of XGB model. The x-axis represents the magnitude of the SHAP values, indicating the extent of impact on the prediction. The y-axis lists the features, indicating the factors under consideration. Green and red bars represent the positive and negative impact of features, respectively. Features that push the prediction higher are shown in green, while those reducing the prediction are depicted in red.

Supplementary Material 6: Calibration curves of three modified baselines

| **Modfied Baseline sCr 1** | | | | | **Modfied Baseline sCr 2** | | | | | **Modfied Baseline sCr** | | | | |
| --- | --- | --- | --- | --- | --- | --- | --- | --- | --- | --- | --- | --- | --- | --- |
| 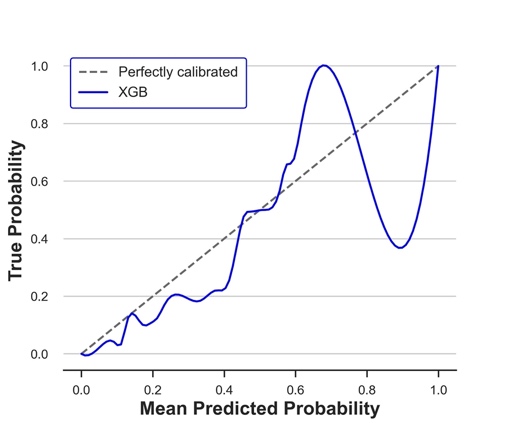  Figure S2 (1). Calibration curve of the XGB with Modified Baseline sCr 1 | | | | | 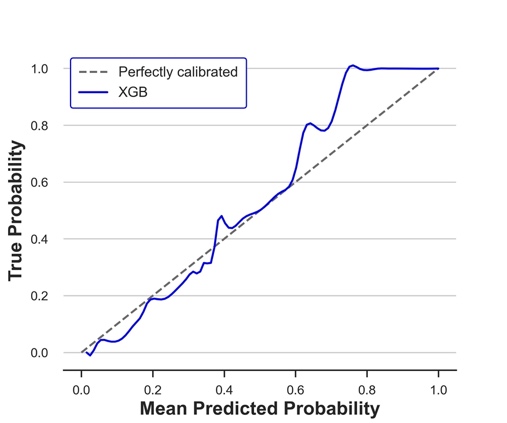  Figure S2 (2). Calibration curve of the XGB with Modified Baseline sCr 2 | | | | | 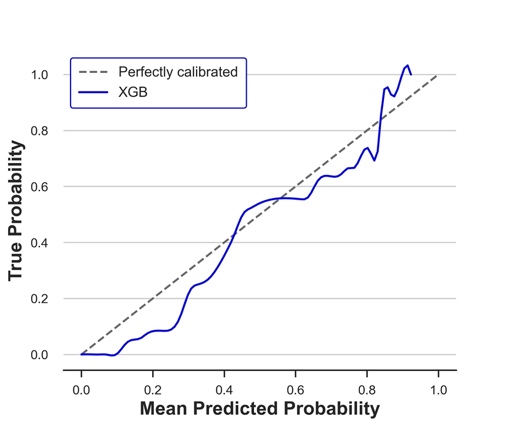  Figure S2 (3). Calibration curve of the XGB with Modified Baseline sCr 3 | | | | |
|  |  | **XGB** |  |  |  |  | **XGB** |  |  |  |  | **XGB** |  |  |
| *ECE* |  | 0.0772 |  |  | *ECE* |  | 0.0670 |  |  | *ECE* |  | 0.0726 |  |  |
| *Brier Score* |  | 0.1213 |  |  | *Brier Score* |  | 0.1231 |  |  | *Brier Score* |  | 0.1000 |  |  |
| *Slope* |  | 1.2056 |  |  | *Slope* |  | 1.2341 |  |  | *Slope* |  | 1.10970 |  |  |

*Abbreviations: sCr, serume creatinine; XGB, eXtreme Gradient Boosting*
